# Engineered Fat Graft Enhanced with Adipose-Derived Stromal Vascular Fraction Cells for Regenerative Medicine: Clinical, Histological and Instrumental Evaluation in Breast Reconstruction

**DOI:** 10.3390/jcm8040504

**Published:** 2019-04-12

**Authors:** Pietro Gentile, Donato Casella, Enza Palma, Claudio Calabrese

**Affiliations:** 1 Department of Surgical Science, Plastic and Reconstructive Surgery Unit, University of Rome Tor Vergata, 00133 Rome, Italy; 2 The Oncologic and Reconstructive Surgery Breast Unit, Oncology Department, Careggi University Hospital, 50134 Florence, Italy; donatocasella@gmail.com (D.C.); enzapalma80@gmail.com (E.P.); claudiocalabrese.it@gmail.com (C.C.); 3 Department of Oncologic and Reconstructive Breast Surgery, “Breast Unit Integrata di Livorno, Cecina, Piombino, Elba, Azienda USL Toscana nord ovest”, 50132 Livorno, Italy; 4 Breast Surgical Oncology Unit, General Hospital, 41125 Modena, Italy; 5 San Rossore Breast Unit, 56122 Pisa, Italy

**Keywords:** Adipose-derived stem cells, ASCs, Breast augmentation fat graft, Engineered fat graft

## Abstract

The areas in which Stromal Vascular Fraction cells (SVFs) have been used include radiotherapy based tissue damage after mastectomy, breast augmentation, calvarial defects, Crohn’s fistulas, and damaged skeletal muscle. Currently, the authors present their experience using regenerative cell therapy in breast reconstruction. The goal of this study was to evaluate the safety and efficacy of the use of Engineered Fat Graft Enhanced with Adipose-derived Stromal Vascular Fraction cells (EF-e-A) in breast reconstruction. 121 patients that were affected by the outcomes of breast oncoplastic surgery were treated with EF-e-A, comparing the results with the control group (*n* = 50) treated with not enhanced fat graft (EF-ne-A). The preoperative evaluation included a complete clinical examination, a photographic assessment, biopsy, magnetic resonance (MRI) of the soft tissue, and ultrasound (US). Postoperative follow-up took place at two, seven, 15, 21, 36 weeks, and then annually. In 72.8% (*n* = 88) of breast reconstruction treated with EF-e-A, we observed a restoration of the breast contour and an increase of 12.8 mm in the three-dimensional volume after 12 weeks, which was only observed in 27.3% (*n* = 33) of patients in the control group that was treated with EF-ne-A. Transplanted fat tissue reabsorption was analyzed with instrumental MRI and US. Volumetric persistence in the study group was higher (70.8%) than that in the control group (41.4%) (*p* < 0.0001 vs. control group). The use of EF-e-A was safe and effective in this series of treated cases.

## 1. Introduction

The popularization of the fat grafting concept has resulted in greater patient demand for breast augmentation that is based on Engineered Fat Graft Enhanced with Adipose-derived Stromal Vascular Fraction cells (EF-e-A) concept. The immediate gratification and the absence of breast implants have become powerful motivators for the patient to request this regenerative surgery.

Adipose-Derived Stem Cells (ASCs) are localized in the Stromal Vascular Fraction (SVF) of subcutaneous adipose tissue [1], which has a heterogeneous mesenchymal cell set [2,3]. When the Stromal Vascular Fraction cells (SVFs) are seeded into a culture, a subset of elongated cells begins to adhere to the tissue culture plastic ware. These cells can be further purified while using a combination of washing steps and culture expansion, with media that are similar to the ones used for bone marrow MSCs, in order to deplete most of the hematopoietic cell population from the SVF cells and differentiate them into classical mesodermal tissues (like bone, fat, and cartilage) after isolation and under specific stimuli [4,5]. They can further hold clinical potential in relation to osteogenesis [6], vasculogenesis [7], and other neuronal repair models [8].

During the last five years, an increasing number of publications have been reported on the translational use of ASCs. The areas in which ASCs and SVFs have been used include radiotherapy based tissue damage after mastectomy [9,10], breast augmentation [11], calvarial defects [12], Crohn’s fistulas, and damaged skeletal muscle [13]. 

All these cell types can be found in the mixed cell population referred to as adipose-derived regenerative cells (ADRCs), which are sometimes also referred to as stromal vascular fraction [14]. ADRCs might improve tissue outcomes by increasing vascularity and through the secretion of growth factors that improve tissue survival. 

Some authors have published randomized clinical trials using cells assisted lipotransfert (CAL) [11] with favorable and unfavorable results. However, they employed different methods of cell obtainment, isolation, and preparation in different clinical settings [15,16,17].

Previously, Cervelli et al. [18,19,20] published works on the use of fat graft according to the Coleman technique [21,22] enhanced with platelet rich plasma (PRP) in plastic surgery [18] and in lower chronic extremity ulcers [19]. Now, the authors present their experience using regenerative plastic surgery with the EF-e-A in breast reconstruction. 

The breast fat grafting technique has been used for many years and it has become rapidly popular, especially in plastic and oncoplastic surgery to reduce injury effects [23,24]. Fat graft can also complete others breast reconstruction techniques improving the cosmetic result in conservative treatment followed by radiotherapy [25].

During breast surveillance any tissue modification needs to be well known to avoid any misleading interpretation between benign and malignant growth. Postsurgical and radio-induced changes are the most common causes of short-term follow up or inconsistent needle biopsy assessment. 

Cell assisted lipotransfert was used in primary breast augmentation [26] and for correcting the sequelae of conservative breast cancer surgeries [14] and congenital deformities [27], but none of these studies were followed by qualitative evaluation of fat graft. In addition, none of the study reviewed explained the concept of “Engineered fat graft”, in which the human body, and in particular, in this study, the breast could be considered as bioreactor for fat grafting injected, favoring neo-angiogenesis that is promoted by SVFs, ASCs, and growth factors release. In fact, ASCs secrete pro-angiogenic factors, such as Vascular Endothelial Growth Factor (VEGF), associate perivascularly with blood vessels, and provide physical Extracellular Matrix (ECM) guidance cues that promote endothelial sprouting [28,29,30]. The concept of “Man as living bioreactor”, was introduced for the first time by Warnke et al. [31] in 2006, and after by Naujokat et al. [32] (2018) and Wiltfang et al. [33] (2016). For the first time, the authors reported, in the present study, the concept of bioreactor as applied to the fat graft and at the breast.

A multimodal imaging approach seems to give the right answer for studying breast tissue modification following surgery both after cancer or purely aesthetic treatment [23].

One of the purpose of the following lines is to describe how modern instrumental techniques of imaging, nowadays available, can show the physiological modifications of the breast tissue, estimate the injected volume fat reabsorption, and evaluate fat replication throughout neo-angiogenesis, in addition to tissue characterization.

Here, the results the authors report suggest the efficacy of EF-e-A and the reported satisfaction of the patients add as a confirmation of the quality of the results.

## 2. Methods

This retrospective observational case-series study was conducted following the principles that were outlined in the Declaration of Helsinki and internationally consented ethics in clinical research [34]. A quality assessment was carried out based on the Strengthening the Reporting of Observational studies in Epidemiology (STROBE) checklist [35]. The Ethics on Research Committee of the University-Hospital, “Careggi” Florence, Italy, with protocol number (Prot. n.) #17199 (approval of 10 May 2008) approved the study protocol, which is where clinical activities were performed. All of the patients received detailed oral and written information about the study, including the risks, benefits, and alternative therapies, and signed an informed consent form before any study procedures.

### 2.1. Patients

Between January 2008 and June 2015, 121 consecutive patients (study group) (SG) diagnosed with outcomes of breast cancer, previously undergoing oncoplastic reconstructive surgery (81 patients affected by the outcomes of mastectomy, 21 patients with outcomes of quadrantectomy, and 19 patients with outcomes of breast reconstruction performed with prosthesis) were treated with EF-e-A for breast reconstruction at the Unit of Breast Surgery of the University of Florence, Italy. In addition, 50 patients (control group 1) (CG 1) were treated with engineered fat graft that was not enhanced with ASCs (EF-ne-A) according to the Coleman technique [21,22] (centrifuged fat graft alone). An additional seven patients that were not treated with any fat graft injection were analyzed (control group 2) (CG2). All of the enrolled patients underwent a full preoperative screening, including a complete clinical examination, photographic assessment, biopsy, appropriate imaging performed by magnetic resonance imaging (MRI), and ultrasound (US). In addition, in the more complex cases, a high-resolution CT scan with three-dimensional (3-D) imaging was performed. Postoperative follow-up took place at 2, 7, 15, 21, 36 weeks, and then annually. 

The average age of patients was 56.24 (range 25–85, standard deviation ± 11.44). Pre-menopausal patients were 46 (38.01%). In 58 patients (48.0%), the outcomes of oncoplastic surgery were in the right breast. 

### 2.2. Inclusion and Exclusion Criteria

**Figure 1:**
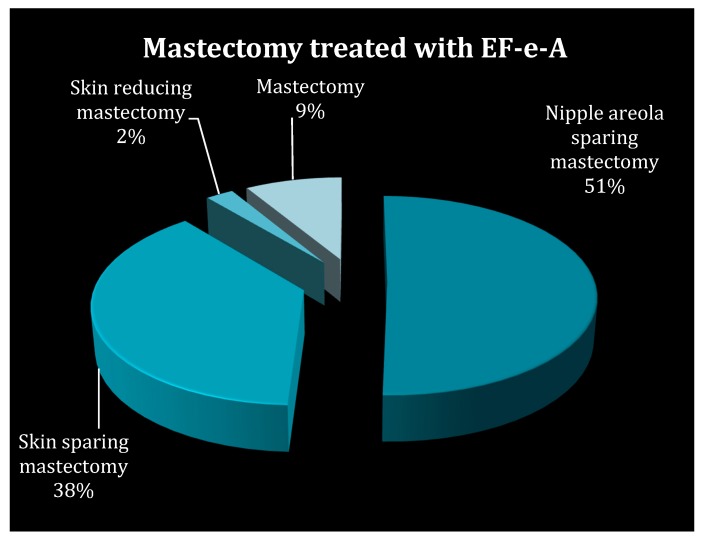
Clinical data of patients underwent oncoplastic surgery. Percentage of: Mastectomy (M); Nipple Areola Sparing Mastectomy (NASM); Skin Sparing Mastectomy (SSM); and, Skin Reducing Mastectomy (SRM).

The inclusion criteria were the following: age 18–75 years old, history of Mastectomy (M), Nipple Sparing Mastectomy (NSM) for a histologically proven Tis-T2N0-N2M0 breast adenocarcinoma in the previous 24 months, Nipple Areola Sparing mastectomy (NASM), Skin Sparing Mastectomy (SSM), Skin Reducing Mastectomy (SRM), breast reconstruction performed by means of a Tissue Expander (TE) temporary breast prosthesis, outcomes of breast reconstruction performed with permanent prosthesis, oncoplastic surgery, mastectomy, active oncological follow-up (according to Italian Association of Medical Oncolgy, AIOM, follow-up schedule guidelines, http://www.aiom.it) with no documented recurrences, and systemic disease at the time of study enrollment. Figure 1 presents the clinical data of all the patients. Mastectomy was performed in the case of multifocal tumor, lobular invasive cancer, lymph-vascular invasion, or when a second conservative operation would have determined an unsatisfactory cosmetic outcome. Additional inclusion criteria in both groups were patients with primary breast reconstruction with contour irregularities, BMI between 20 and 35 kg/m^2^, sufficient fat in the abdomen, thighs, flanks, and inner knees regions (site of fat harvest). Radiotherapy, despite being a confounder factor, was not regarded to be an exclusion criterion; however, only patients with grades 1 and 2 in the LENT-SOMA scale [36] were included. A stratified blocked randomization was also done to evenly distribute the patients with radiotherapy [37]. Patients with breast cancer active disease sequelae of breast cancer conservative treatment, smokers, and uncontrolled comorbidities were excluded.

### 2.3. Clinical Data Assessment

Data were prospectively recorded in a database with SQTM® (“Computerized card for the control of the quality of treatment of breast cancer software”) software (CPO, Tourin, Italy). The following characteristics were prospectively recorded in the dataset: demographic data, age, BMI, histological evaluation, surgical and oncological management, surgical complications, time and site of recurrence, adjuvant or neo-adjuvant radiotherapy, and chemotherapy data. All of the therapeutic options were discussed and decided upon by a multidisciplinary team, including a breast surgeon, a plastic surgeon, a pathologist, a radiologist, an oncologist, a radiotherapist, and a psyco-oncologist. The oncoplastic technique, previously applied, was determined by patients’ anatomy, preferences, and tumor location. All of the patients were treated with an oncoplastic approach, when a significant volume excision was followed by a reshaping of the breast parenchyma with volume displacement technique, accompanied by an adequate skin envelope reduction. According to patients’ preferences, contralateral symmetrization was performed during the same operation, when necessary. During the first five years, the patients were followed up every six months by clinical examination and every 12 months by surveillance mammogram and MRI. Abnormal clinical findings were further investigated as appropriate. Since the fifth year, follow up was carried out yearly. Recurrences were documented by clinical examination, radiological tests, and/or pathological assessment. Local and distant recurrence rates were the primary outcomes and they were evaluated as the oncological safety outcome.

### 2.4. Fat Grafting Preparation

#### 2.4.1. Engineered Fat Graft Enhanced with Adipose-derived Stromal Vascular Fraction cells (EF-e-A)

**Figure 2:**
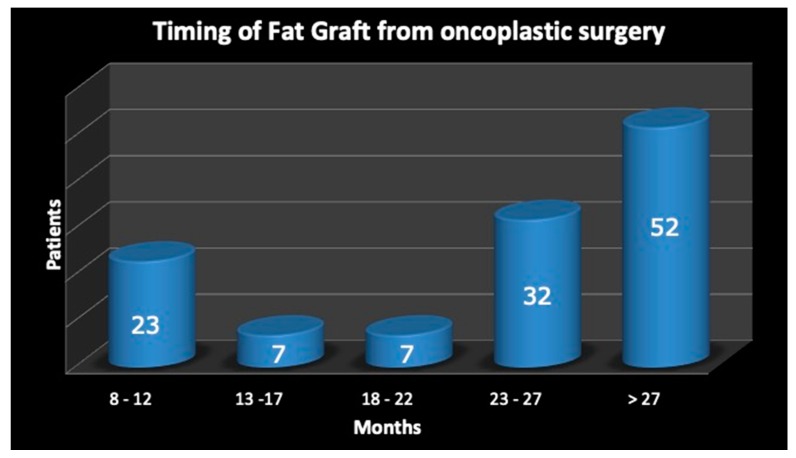
The timing of fat graft, performed after oncoplastic surgery.

The cell and tissue preparation procedure mainly exhibited two phases. Phase one start with a syringe liposuction (715.4 mL average in all patients—range 250 mL/1080 mL) in the abdominal region using 3 mm cannulas. Maintaining aseptic technique, the plunger of the 60 mL-syringe was removed and the tip was closed with a cap. Half of the lipoaspirate (234.46 mL average) was placed into the tissue collection container of the Celution™ 800/CRS System (Cytori Therapeutics Inc., San Diego, CA, USA). Through a wash cycle, blood and free lipid were removed from the tissue and the Celase™ 835/CRS Reagent was added to enzymatically digest the tissue, which released SVFs. After additional wash and centrifugation cycles, 4–5 mL of the SVFs suspension was extracted from the system. In the second phase, the remaining part of lipoaspirate was added to the tissue collection container and a washing step was automatically carried out. Once completed, the 4–5 mL of SVFs suspension was added and then mixed with the washed fat graft, resulting in approximately 429.61 mL (range 60 mL/620 mL) of SVF-enhanced fat tissue for grafting. This newly processed cell-enhanced fat graft typically consists of 25–30% of water, which will be reabsorbed by the body in the post-operative period. This overall process was controlled through automated sensors and processing algorithms that ensure the standard handling of the tissue and cells, and the process was completed within 160 min. The SVF-enhanced fat graft, which the authors called “Engineered Fat Graft Enhanced with Adipose-derived Stromal Vascular Fraction cells” was transferred into 10 mL syringes and aseptically re-injected into the patient using specific micro-cannulas for implantation. The timing of fat graft, performed after oncoplastic surgery, is reported in Figure 2.

#### 2.4.2. Advantages of ASCs/SVFs-Supplemented Fat Graft in EF-e-A

One of the main reasons why the authors used this technique of Engineered Fat Graft Enhanced with Adipose-derived Stromal Vascular Fraction cells is that there was less reabsorption of the fat graft injected when compared with a non-enhanced fat graft. The reabsorption rate, as reported over the first year, is highly variable, represented by 37% when SVFs-enhanced fat graft was used and 61% for not enhanced fat graft [38]. To prevent reabsorption, it was crucial to perform each step of the procedure carefully, paying close attention to the technical details. The donor site region (abdomen) was infiltrated with a cold saline solution containing 1 mL of adrenaline per 500 mL of saline solution without lidocaine or carbocaine to reduce the bleeding during the procedure. An inverse relationship has been observed between the blood amount in the lipoaspirate and the viable number of adipocytes [39]. The adipose tissue was removed after 5 min while using a 3-mm-diameter cannula and a 60-cc Toomey syringe. The authors re-injected the EF-e-A using specific micro-cannulas (1.5 mm in diameter) for implantation. 

#### 2.4.3. Engineered Fat Graft not-Enhanced with Adipose-Derived Stromal Vascular Fraction Cells (EF-ne-A)

Engineered Fat Graft not Enhanced with Adipose-derived Stromal Vascular Fraction cells was performed according to the Coleman technique [21,22]. The fat tissue was harvested from the abdominal region using some specific cannulas. Maintaining asepsis, we took the plungers off the syringes; after closing them with a cap, we positioned them flat in the sterile centrifuge. The syringes were processed for 3 min at 3,000 rpm. This procedure obtained purified fat tissue, preserving the integrity of the adipocytes but separating the fluid fat portion from the serous bloody part. The purified centrifuged fat was put in 1-mL syringes and then aseptically reinserted while using the specific micro-cannulas for implanting. Neither of SVFs nor ASCs addition was performed.

### 2.5. Surgical Technique

The area that was destined to receive the fat graft was determined based on the necessary corrections. Based on this, the harvested material has been implanted for breast augmentation prevalently into three areas: inferior breast rim, superior, and inferior region of the areola and in the superior lateral quadrant. 

The fat injection was performed while using the "Gentle technique” [39] based on a slow and gentle injection implanting linear deposits of fat graft in the suprafascial, retroglandular and intraglandular space [39,40]. For this reason, the EF-e-A was implanted in multiple tunnels with slow and controlled movements through different entrances (inframammary fold, supero and infero-external quadrant, supero and infero-internal quadrant, periareolar) to underline the importance of a non-traumatic procedure to maximize the integrity of the grafted fat and to maximize the contact surface between the lipoaspirate and the host’s capillaries [38,39]. The diffusion of the nutrients from neighboring capillaries is essential in adipocyte survival and it favors their integration with the surrounding tissue [38,39]. The incisions were closed with a 5-0 nylon suture and no compressive bandage was applied. 

### 2.6. Breast Volumetry

The patients were MRI scanned with previous injection of gadolinium-contrast. They were scanned again at six and 12 months after the EF-e-A injection and then annually, with the purpose of determining the breast volume. A 1.5 Tesla scanner (Hitachi, MS, Echelon Oval, Tokyo, Japan) was employed with 3 mm thick slices. OsiriX software, 32 bits, free version (Pixmeo, Bernex, Switzerland) was utilized to calculate breast volume. Two calculations were completed per exam and the average determined was taken as the final breast volume.

### 2.7. Clinical Evaluation Method

Two methods for the clinical evaluation of outcomes were used: Team-evaluation and patient self-evaluation. The Team evaluation was an evaluation method that was based on clinical observation, using a scale of six values (excellent, good, discreet, enough, poor, inadequate). The patient-based self-evaluation used the same six values that are mentioned above. The factors/variables, which were taken into account, were pigmentation, vascularization, pliability, thickness, itching, and pain.

### 2.8. Histological and Bio-Molecular Cellular Characterization

As secondary end-points of the study, a cellular characterization and a histological evaluation were conducted to compare the two different fat grafting procedures on a translational basis. 

#### 2.8.1. Characterization of SVF-Derived Stem/Progenitor Cells

The quality and quantity of SVF-derived stem/progenitor cell populations were characterized by specific immunohistochemistry markers for the following mesenchymal stem cell-defining surface markers: CD31, CD34, CD45, CD90, and CD105. The analysis was performed, for comparison, on lipoaspirate samples before any handling (CG2), on the samples of EF-e-A in SG patients and also on EF-ne-A from CG1 patients. Such a characterization was performed on the first five patients of both groups (SG and CG1), to evaluate the different cellular nature of transplanted adipose tissue between the two groups. The values were expressed as number of cells per mL.

#### 2.8.2. Histological Analyses of Mastectomy Flap Biopsies

The core biopsies of the mastectomy flaps previously submitted to fat grafting were obtained from six patients, three from SG and three from CG1. Biopsies were all performed in a time span between 12 and 15 months after fat grafting. The biopsies were assessed and studied at the cell laboratories of the University of Modena, Italy.

Five-µm paraffin sections were stained with Hematoxylin and Eosin for histological analysis. Three slides for each patient were carefully analyzed 100× and 400× high power field to investigate the amount of adipose tissue (AT). A scoring system was elaborated to quantify the percentage of normal AT and and/or the presence of two other main features, namely fat reabsorption areas and fat connective tissue (CT) areas. A percentage of 25, 50, 75, or 100 was assigned to the area that was covered by each type of tissue identified as a proportion of the entire area of every single slide analyzed. Subsequently, the average score of all slides for the six patients belonging to SG and CG1 was calculated for the three histological features identified.

In parallel, Sirius Red staining was introduced to evaluate the fibrotic areas inside all of the transplanted tissue areas. Five-µm paraffin sections were dehydrated and stained with Carazzi Haematoxylin and then Sirius red (0.1% in picric acid) [41].

Angiogenesis was also investigated by means of immunohistochemistry using anti-CD31 antibody (Ab) and anti-αSMA Ab, as reported in literature [5,42]. CD31 is an endothelial marker for both mature and immature vessels, and α-SMA is as pericytic marker that stains the extra endothelial part of mature vessels [42]. Five-µm paraffin sections were dehydrated and stained with mouse anti-human CD31 Ab (1:50) or rabbit anti-human αSMA Ab (1:200) while using a goat anti–mouse or goat anti-rabbit biotinylated secondary Ab (1:200) and an avidin-biotin-horseradish peroxidase detection system. The analyses were performed using 100× magnification. Scoring was performed by counting CD31+ vessels/100× high power field and counting α-SMA + vessels/100× high power field (*n* = 10/each specimen). 

### 2.9. Statistical Analysis

Standard descriptive statistics were used to summarize data. The Kruskal–Wallis rank test for continuous variables and Pearson’s Chi square test (or Fisher’s exact test when appropriate) for categorical variables performed a comparison of the clinical and biological characteristics between the three groups of patients. Logistic regression was used to investigate which factors were associated with recurrence events, either loco-regional or systemic. The independent variables of interest were: surgical technique, age above 50 years old, lymph-vascular invasion, oncological stage, adjuvant or neo-adjuvant chemotherapy, adjuvant radiotherapy, and adjuvant hormone therapy. Multiple logistic regression analyses were performed to simultaneously account for several confounding variables and included all the variables of interest. A comparison between SG and CG1 was done with Student’s *t*-test or Mann–Whitney for breast volumetry, fat graft volume, time of follow-up, cell counting, question of the self-assessment questionnaire, and surface markers expression. The data is expressed by mean (range and standard deviation), median (range), and percentages. For cellular characterization, values are reported as an average number of the samples analyzed. For histological parameters assessment, data are expressed as mean values +/− standard error of the mean (SEM). A two tailed *p*-value of less than 0.05 was considered to be significant. 

## 3. Results

### 3.1. Clinical Assessment

The transplantations of EF-e-A and EF-ne-A were successfully performed in all cases. In 72.8% (*n* = 88) of breast reconstruction treated with EF-e-A (SG), we observed a restoration of the breast contour and an increase of 12.8 mm in the three-dimensional volume after 12 weeks, which was only observed in 27.3% (*n* = 33) of patients in the control group 1 (CG1) that was treated with EF-ne-A. 

All of the patients treated (SG and CG1) were satisfied with the resulting texture, softness, and contour. In both groups (SG and CG1), the vast majority of patients were satisfied with the results of fat grafting (*p* = 0.603) would available undergo the fat grafting procedure again (*p* > 0.999) and would recommend the fat grafting procedure to a friend (*p* = 0.546). When the patients were allowed to freely give a score to their cosmetic result (self-evaluation), the scores ranged from 3 to 6 in control group 1 and from 1 to 4 in study group (*p* = 0.075). These results show a strong trend in the patients of the study group to be more pleased than the patients in the control group. The analysis of the satisfaction assessment questionnaire showed that all patients in both groups would choose to undergo breast reconstruction with fat graft, and they were sufficiently informed about this procedure. 

**Figure 3:**
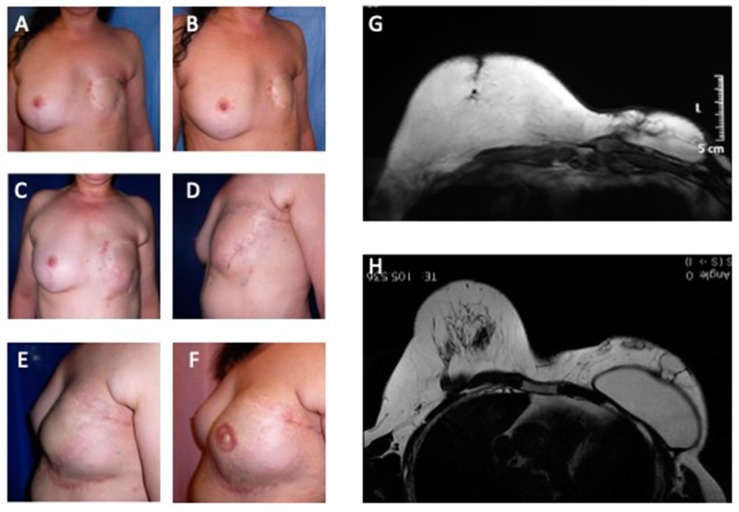
Analysis of study group’s patient affected by outcomes of tram-flap failure. (**A**) Pre-operative in frontal view after outcomes of mastectomy in left breast; (**B**) Pre-operative in ¾ right view after outcomes of mastectomy in left breast; (**C**) Post-operative in frontal view after six months from 1st Engineered Fat Graft Enhanced with Adipose-derived Stromal Vascular Fraction cells (EF-e-A) injection in left breast; (**D**) Post-operative in ¾ left view after six months from 1st EF-e-A injection in left breast; (**E**) Post-operative in lateral left view after nine months from 2nd EF-e-A injection in left breast and implant of prostheses; (**F**) Post-operative in lateral left view after three months from nipple areola complex reconstruction; (**G**) Pre-operative MRI image of patient, before the prostheses implant and six months from 1st EF-e-A injection in left breast (condition referred at picture C,D); (**H**) Post-operative MRI image of patient, after the prostheses implant and nine months from 2nd EF-e-A injection in left breast (condition referred at picture E).

When satisfaction was evaluated through a visual analogue scale (VAS), patients of both groups were similarly satisfied (*p* = 0.52). Initially, the five-peer analysis showed disagreement in the pair-to-pair comparison and in the general comparison, with low values of the kappa coefficient. Accordingly, changing the five subsets into three (worsened (−1), nothing changed (0), and improved (+1)), surgeons agreed to a minor degree (kappa = 0.131, confidence interval = 0.020; 0.242). Figure 3 showed patients that were categorized as showing “improvement” by all peers. When computing the new scores, patients in the study group and in the control group 1 received the respective scores (average) of 3.1 and 2.5 (*p* = 0.60) and, therefore, were regarded as presenting similar improvement.

### 3.2. Imaging Assessment

#### 3.2.1. Breast Surveillance

**Figure 4:**
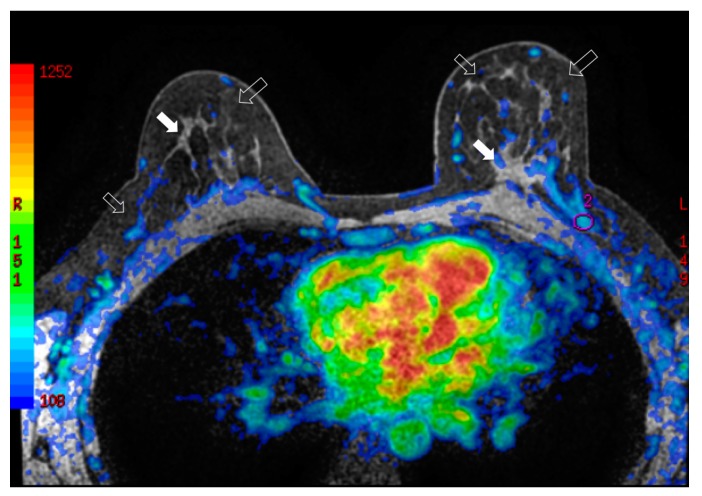
Bilateral three-dimensional MRI T1-weighted spoiled turbo gradient echo image after contrast media administration (colored superimposed image). Using a special fat saturation and separate shim volumes on each breast, VIBRANT sequence (Volume Imaging for Breast Assessment) allows axial acquisition with an excellent separation of glandular tissue (white arrows) from fat tissue (white empty arrows). The velvet region of interest shows how the vascularity of the left breast compared to the contralateral breast after fat grafting is increased.

During breast surveillance, any tissue modification needs to be well known to avoid misleading interpretation between benign and malignant growth. To better identify the glandular and adipose tissues, a multimodal imaging approach seems to give the right answer for studying breast tissue modification following fat grafting. Figure 4 showed the physiological modifications of the breast tissue and evaluate fat replication throughout neo-angiogenesis. In particular, Figure 4 demonstrates how the vascularity of the left breast is increased when compared to the contralateral breast after fat grafting.

#### 3.2.2. Fat Reabsorption

Transplanted fat tissue reabsorption was analyzed with instrumental MRI and ultrasound. The volumetric persistence of fat graft in the study group was higher (70.8%) than that in the control group 1 (41.4%) (*p* < 0.0001 vs. control group 1). When compared with breast reconstruction using EF-ne-A, reconstruction with EF-e-A showed a smaller rate of fat reabsorption.

**Figure 5:**
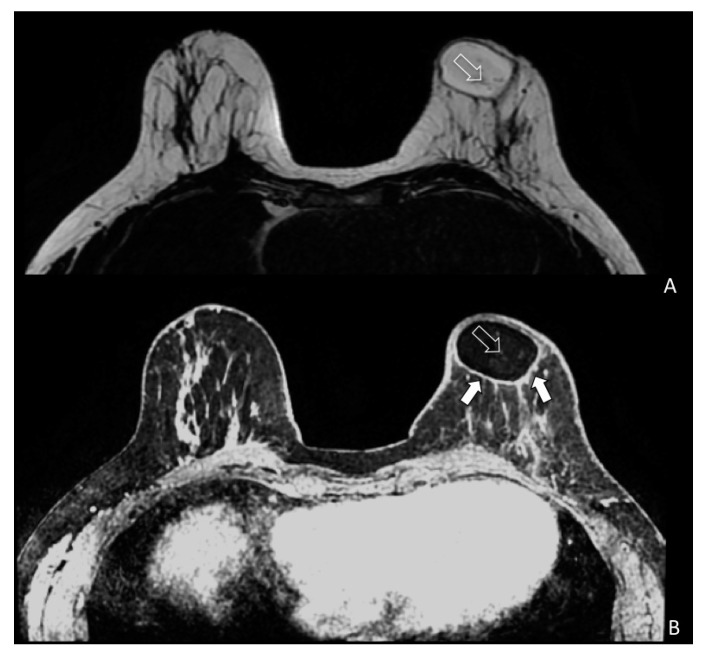
Magnetic resonance imaging (MRI) of a patient (SG) treated with EF-e-A. (**A**) Axial MRI T2-weighted turbo spin echo image of both breasts. Fat is imaged with hyper-intense signal and glandular tissue with the matrix is characterized by hypo-intense signal. In the left breast is showed an oval area of hyper-intense signal representing the fat grafting. Very small vessels are depicted in the area (white empty arrow); (**B**) Bilateral three-dimensional T1-weighted spoiled turbo gradient echo image after contrast media administration VIBRANT sequence showed contrast uptake of the glandular tissue and of the fat graft boundary (white arrows). In this sequence, fat graft is characterized by hypo-intense signal due to the fat saturation pulse. In the fat graft, small vessels characterized by contrast media uptake are confirmed (white empty arrow).

Cyst formation, micro, macro—calcifications and cytosteatonecrotic areas (Figure 5A,B) were detected by MRI. Fat necrosis was present in four patients in the study group and in one of patients in the control group (*p* = 0.103). Fat necrosis was surgically removed and the pathological findings confirmed this diagnosis for three patients. One patient was observed and the ultrasound follow-up showed no need for intervention. In the long-term follow-up, adverse events, like infections and skin necrosis, were not observed in both groups. 

### 3.3. Local Recurrence and Metastases

Loco-regional recurrences and metastases were observed. The median follow up was 73.1 months; two patients were lost at follow-up (1.66%). Local recurrences (LR) were observed in seven patients (5.8%) in an average time of 33.3 months (range 16–61). The risk of local recurrence was significantly higher in the group of patients with lymph-vascular invasion and with high grade (G). Five patients (4.15%) developed distant metastases, in an average time of 36.1 months after the treatment (range 18–68). 

SG presented one local recurrence, which was represented by an axillary lymph-node. Three systemic recurrences were also recorded in this group. Two of them were bone metastases, while the remainder was a case of pulmonary plus liver metastases. Disease free survival (DFS) was 19, 22, and 25 months after the last reconstructive stage and 37, 34, and 38 months after NSM. Among control group 1 (CG1) patients, there were three local recurrences and two systemic recurrence events; one patient had both a local and systemic recurrence. Local recurrences were a mastectomy flap nodule documented 28 months after NSM and 15 months from reconstruction with EF-ne-A (centrifuged fat) and two loco-regional axillary lymph-nodes documented 24 and 27 months from fat graft and 32 and 40 months from NSM, respectively. 

Systemic recurrences were represented by n.1 pulmonary and n.1 brain metastasis, which occurred 14 and 24 months from the second stage and 22 and 32 from NSM, respectively. 

In the control group 2 (CG2), one local recurrence, which was located in the nipple, and two systemic recurrences, one bone, and one bone plus liver, were reported. The DFSs are 11, 13, and 9 months from the second stage and 23, 34, and 26 months from NSM, respectively. 

Multivariate analysis showed that, among the oncological variables included, there were no significant risk factors, which would lead to a recurrence event, either loco-regional or systemic. In particular, the adopted technique (enhanced and not enhanced fat graft) did not represent a significant risk factor for recurrences (Table 1).

### 3.4. Histological Assessment

The evaluation of the SVF-derived cell population showed a consistent difference in the stem/progenitors cells amount in the transplanted specimens when considering that the standard centrifuged fat comprises half of the adipose derived regenerative cells (ADRCs) population than the enhanced fat (Table 2). 

**Figure 6:**
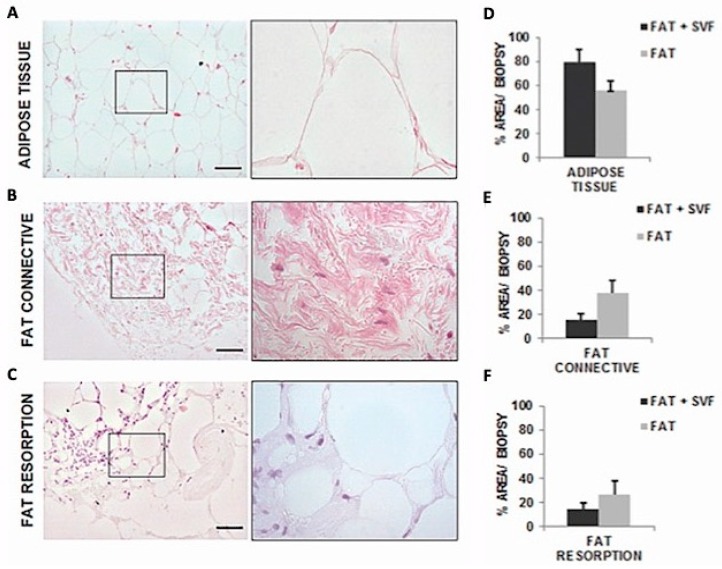
Images of Hematoxylin and Eosin stained sections (100× magnification and inset 400×) with tissue distribution for the three identified histological features within the biopsies sections. In both groups (SG and CG1) Adipose Tissue (AT) (**A**) were characterized by round shaped adipocyte without any abnormality. Both groups presented connective tissue (CT) (**B**), characterized by fibroblastoid cells surrounded by oriented collagen fibers. Fat reabsorption area was an anomalous AT composed by inflammatory cells infiltrating the lobules of adipocytes and associated by small/medium size cysts delimited by polymorphonucleated cells (**C**). On average, these adipocytes had higher diameter in size versus the normal adipocytic cells. (**D**–**F**) showed the comparison of score system results between the two groups in the attempt to investigate the contribution of SVF in the maintenance of AT and CT-reabsorption reduction.

Core biopsies histological characterization of fat grafted flaps showed the presence of three main areas: Adipose Tissue (AT), connective tissue (CT), and fat reabsorption. The average area that is covered by AT is 79.17% (±10.62 SD) for SG versus an average of 56.25% (±7.83 SD) in CG1, corresponding to a fold of increase (FI) of 1.4 in AT. On the contrary, CT is less present in SG, 15.28% (±5.42 SD) versus CG1, 37.5% (±10.56 SD), with a fold of decrease (FD) of 2.5. Down the same line, it is possible to show an increase of fat reabsorption areas in CG1, with an average of 14.35% (±5.29 SD) for SG versus 26.63% (±11.13 SD) for CG1, corresponding to a FI of 1.9 in tissue reabsorption. These results, by the way, did not reach a statistical significance by the t-test due to the low number of biopsies analyzed (Figure 6). 

As for the assessment of fibrotic areas within AT, the level of Sirius red positivity was lower than 2% in both SG and CG1, 1.28% (±0.28 SD) and 1.85% (±0.25 SD), respectively. However, this parameter significantly increased in the region of fat reabsorption with a higher level in CG1, 8.52% (±0.88 SD), rather than SG, 6.49% (±0.95 SD), specimens. 

**Figure 7:**
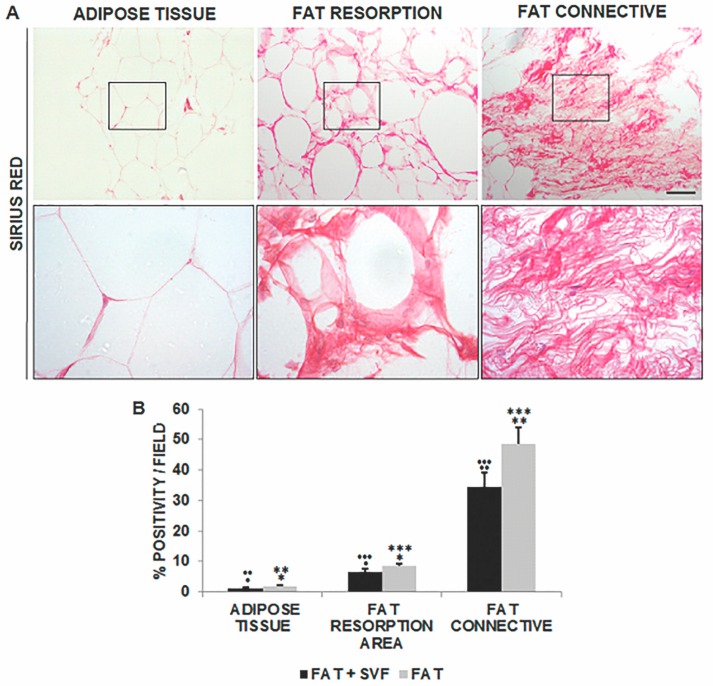
Images of Sirius red stained sections (100× magnification and inset 400×) displaying the histological features of AT (**A**, left panel and inset), reabsorption area (**A**, middle panel and inset) and CT (**A**, right panel and inset). The intense red color marked the dense connective areas inside the samples, while the other parenchymal tissue appeared rose in color. This latter feature (rose) has been excluded from the analyses because it is not indicative of collagen deposition. Thus, Sirius red quantification was performed for each of the distinct fat features/areas in the two groups. Data were reported in (**B**). The statistical symbols *, **, *** and **•**, **••**, **•••** indicate the maximum value and outliers. *, **•** are maximum values; **, **••** are two values above the limit; ***, **•••** are three values above the limit (outliers).

While taking CT into account, Sirius red staining was highly predominant in the CG1 specimens with approximately 50% of fibrotic tissue within the stained areas, 48.70% (±5.24 SD). Lower levels of Sirius red stained fibrosis could be detected in SG areas, 34.42% (±4.93 SD), suggesting the positive contribution of SVF in the maintenance of fat after breast reconstruction surgery (Figure 7).

**Figure 8:**
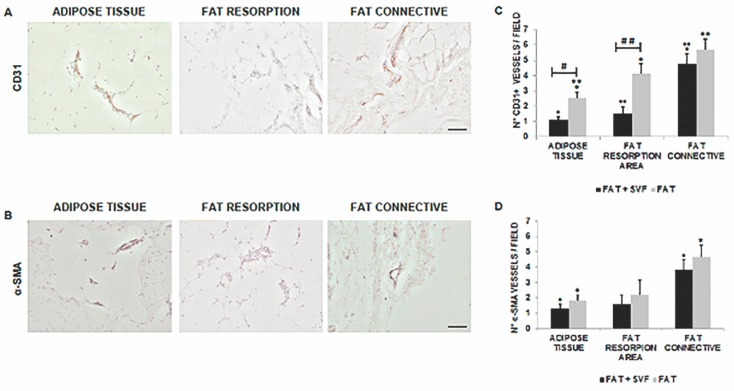
(**A**,**B**) displayed representative 100× magnification images of AT, fat reabsorption area and CT stained for CD31 (brown color; DAB as chromogen) and α-SMA (black color; DAB-Nickel as chromogen) within a mastectomy flap biopsy. (**C**,**D**) show the results of comparison between the two groups. The statistical symbols *, **; •, •• and #, ##, indicate the maximum value and differences between study and control group. *, • are maximum values; **, •• are two values above the limit; # indicate marked difference between the study group and the control group; ## very marked difference between the study group and the control group.

As for angiogenesis, collectively, both CD31+ and α-SMA+ vessel density for each of the analyzed fat features/areas were higher for the G2 specimens versus G1 samples (Figure 8). The scoring was reported in Figure 8C (for CD31+ vessel) and three-dimensional (3D) (for α-SMA+ vessel). 

In particular, when comparing AT and the fat reabsorption areas, the differences for CD31 stain between the two groups were statistically significant (*p* = 0.002 and *p* = 0.003, respectively), while the same was not found for α-SMA (*p* > 0.05). Similarly, the amount of vessel density (CD31+ and α-SMA+) of connective associated fat tissue relative to the CG1 samples was again higher than the SG samples but not statistically significant.

### 3.5. Histological and Imaging Assessment in Combined

**Figure 9:**
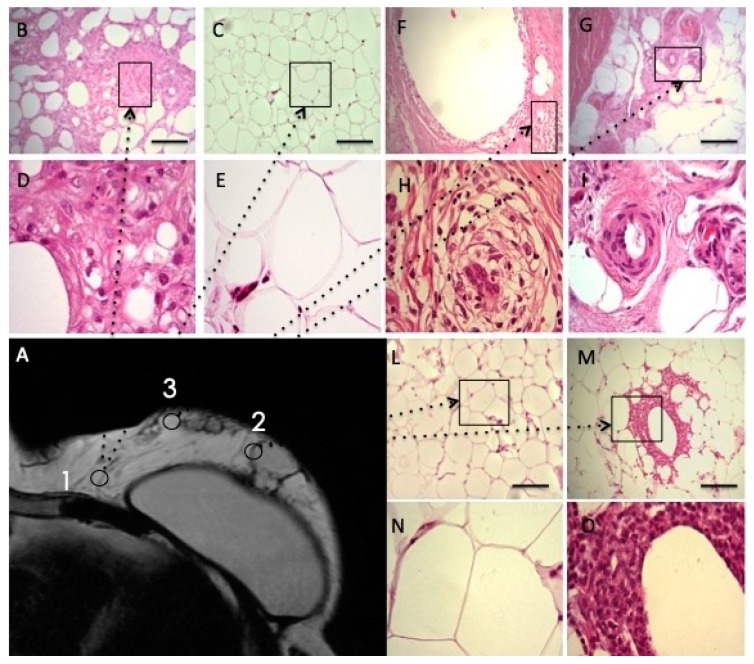
Biopsies analyses, one year later, the enhanced fat graft’s injection. (**A**) MRI image. The point 1 show the biopsy site, harvested on fat graft performed into inner quadrant of the breast close to the chest wall. Histological analysis of the biopsy reported in Images (**B**–**E**) as Hematoxylin and Eosin stained sections (100× magnification and inset 400×) showed tissue that was characterized by round shaped adipocyte without any abnormality. Normal fat tissue and adipocytes were indicated by arrows. The point 2 show the biopsy site, harvested on fat graft performed into more outer quadrant of the breast. Histological analyses of the biopsy, as reported in Images (**L**–**O**) as Hematoxylin and Eosin stained sections (100× magnification and inset 400×), have reported fibrotic tissue substitution with neo-vascularized regenerative fat (arrow). Therapeutic angiogenesis at the regenerative margin was showed (arrow). The point 3 show the biopsy site, harvested on fat graft performed into medial-outer quadrant of the breast. Histological analysis of the biopsy reported in Images (**F**–**I**) as Hematoxylin and Eosin stained sections (100× magnification and inset 400×) showed fibrotic partially remodeled tissue (arrow). Poor neo-angiogenesis with the persistence of remodeling areas was also reported (arrow). Regenerative process “in progress”.

To better understand the analyses of enhanced fat graft injected in the breast, the authors reported the biopsy’s histological evaluation in Figure 9 (Figure 9B–O); one year later the enhanced fat graft injection and they indicated the three different points of biopsy harvest on the Magnetic Resonance Image (Figure 9A). Histological analysis of the biopsy, harvested in the inner quadrant of the breast (Figure 9B–E, point 1 in Figure 9A), showed normal fat tissue characterized by round shaped adipocyte without any abnormality. The biopsy of fat graft performed into a more outer quadrant of the breast (point 2 in Figure 9A) was analyzed in Figure 9L–O reporting the fibrotic tissue substitution with neo-vascularized regenerative fat (arrow) and angiogenesis at the margin (arrow). Histological analysis of the biopsy, which was harvested on fat graft performed into medial-outer quadrant of the breast (point 3 in Figure 9A), is reported in Figure 9F–I showing fibrotic partially remodeled tissue (arrow) and poor neo-angiogenesis.

## 4. Discussion

When compared with breast reconstruction using implants [43], augmentation with SVFs-enhanced fat graft showed lower height but a more natural contour and softness of the breasts [27]. 

In this case series, the supplementation of autologous fat grafts using EF-e-A improved the breast soft tissue volume, when compared to EF-ne-A. The ability of cells to secrete various growth factors that improve survival and increased vascularization, leading to an increased survival of the graft, as shown in the rodent study, could explain the potential benefit of ASCs and SVFs supplementation. 

In fact, ASCs secrete pro-angiogenic factors, such as VEGF, which perivascularly associate with blood vessels, and provide physical Extracellular Matrix (ECM) guidance cues that promote endothelial sprouting [28,29,30]. 

None of the study cited in www.PubMed.gov (six studies in total), searching “Human body as living bioreactor” introduced the concept of “Engineered fat graft”, in which the human body, and in particular the breast, could be considered as a bioreactor for fat grafting injected, favoring neo-angiogenesis that is promoted by SVFs-ASCs, contained in EF-e-A and growth factors release. Warnke et al. introduced the concept of “Man as living bioreactor” for the first time by [31] in 2006, and after by Naujokat et al. [32] (2018) and Wiltfang et al. [33] (2016). In detail, Warnke et al. [31] reported a novel method of repairing a human mandible by in vivo tissue engineering, in which the patients served as his own bioreactor as the exogenously prepared customized mandible replacement was grown inside his latissimus dorsi muscle prior to transplantation to repair the existing defect. Wiltfang et al. [33] and Naujokat et al. [32] reported the world’s first reconstruction of a mandibular discontinuity defect while using a custom-made bone transplant that had been prefabricated in the gastrocolic omentum using tissue-engineering strategies.

Now, for the first time, the authors reported, in the present study, the concept of bioreactor, as applied to the fat graft and at the breast.

In light of this concept, we proposed the chain of events leading to the regeneration of the tissue to be the following: targeting of damaged areas, release of angiogenic and anti-apoptotic factors, followed by the formation of new vessels and oxygenation.

SVFs might indeed improve the fat graft survival and maintenance, which is supported by observations from other surgical procedures, such as maxillofacial surgery for a calvarial defect [12] and breast reconstruction after partial mastectomy with radiotherapy damage [10]. 

The implanted adipose tissue must survive by a simple diffusion mechanism until an active blood supply is re-established. Thus, survival of the graft, particularly of a larger volume graft, is balanced between this process and hypoxia-induced cell death. Therefore, pro-survival factors may promote long term retention and hence the durability of the graft. In an animal study, this effect was achieved by using gene therapy to deliver Vascular Endothelial Growth Factor (VEGF; a potent pro-angiogenic factor) to the graft. This resulted in increased blood vessel density within the graft and a significant improvement in graft retention at 15 weeks. 

SVFs can favour neo-angiogenic vascularization and the fibrogenic activity of fibroblasts that favour adipose tissue survival and three-dimensional organization. When compared to traditional fat grafting, the survival of the graft is more probable and fat necrosis is potentially reduced due to improved vascular development in the implanted area. The results of this study offered an in vivo tissue-engineering approach that provided an optimized microenvironment, supporting the correct architectural adipocyte distribution, better cell-to-cell interaction, adipose tissue survival, and perhaps limited differentiation from SVFs; this could offer early protection from surrounding inflammatory events. Additionally, the early establishment of new micro-capillary networks, which deliver the proper nutrients and oxygen to the implant, might contribute to the improved outcomes that were observed [11]. 

Breast composition is mainly based on a mixture of three tissue types: glandular, fat, and fibrous tissue [41]. Depending on the ratio between these tissues, the breast is classified by the American College of Radiologist in different categories [44] with a different risk of cancer. As is well known, any surgical intervention or radiation therapy increases the risk of fibrous tissue growth with a reduction sensitivity of screening x-ray mammograms. At last but not least, in the case of fat grafting, mammography and breast ultrasound are not able to depict fat tissue quality.

Fat grafting is an important clinical application in the treatment of post-surgical deformities. The simplicity of fat grafting procedures and the absence of a subsequent visible scar prompted an increasing interest for this technique. Adipose-derived stem cells that were obtained from stromal vascular fraction of adult adipose tissue provided exciting perspectives for regenerative medicine and surgery due to adipogenesis and angiogenesis [44]. Angiogenesis is the formation of new blood vessels and can be induced by tissue wound, inflammation, and tumor growth [28,29,42]. Hypoxia-inducible factor (HIF) is a transcription factor that responds to changing intracellular oxygen concentration. During hypoxia, HIF accumulates and it is transported to the nucleus where it induces the expression of numerous target gene products. Secreted growth factors induce signaling pathways, resulting in endothelial cell proliferation, increased vascular permeability, and cell migration. Extracellular matrix proteases and regulators induce tissue matrix remodeling in preparation for the migration of endothelial cells from existing vessels to form new tubing. Tissue wounding, ischemia, or inflammation recruit macrophages and bone marrow-derived inflammatory cells to wound areas and they secrete a similar panel of proteins to induce angiogenesis [5,45,46,47].

The role that is played by the adipose tissue and their secretions, ie adipokines, is beginning to be recognized. Plasma adipokine levels, which are modulated during obesity, could have “remote” effects on mammary carcinogenesis [48]. Breast cancer cells are surrounded and locally influenced by an adipocyte microenvironment, which is probably more extensive in obese people [48]. In a study of Delort et al. [48], leptin appears to be strongly involved in mammary carcinogenesis and it may contribute to the local pro-inflammatory mechanisms, especially in obese patients, who have increased metastatic potential and greater risk of mortality. Really, the percentage of recurrences that were reported in the present work, in particular in the control group 2 (CG2) not treated with fat graft injection, in which all of the patients were affected by obesity, suggests the crucial role of obesity in breast cancer. In fact, in a group of seven patients (CG2), three recurrences (two systemic and one local) were recorded, as compared with four recurrences (three systemic and one local) in study group (SG) that was composed by 121 patients and five recurrences (two systemic and three local) in control group 1 (CG1) composed by 50 patients. Obesity is a well-known risk factor of breast cancer in post-menopausal women that also correlates with a diminished therapeutic response [49]. While the precise correlation between obesity and breast cancer remains to be determined, a recent study [50] suggests that adipose tissue and adipose stem cells influence breast cancer tumorigenesis and tumor progression. Strong et al. [50] reported that ASCs that were derived from the abdominal subcutaneous adipose tissue of obese subjects (BMI > 30) enhanced breast cancer cell proliferation in vitro and tumorigenicity in vivo. These findings were correlated with changes in the gene expression profile of breast cancer cells after co-culturing with ASCs, particularly in the estrogen receptor-alpha (ESR1) and progesterone receptor (PGR) expression [50]. An analysis of the gene expression profile of the four groups of ASCs revealed obesity induced alterations in several key genes, including leptin (LEP). Blocking estrogen signaling with ICI182.780, leptin neutralizing antibody or letrozole diminished the impact of ASCs that are derived from obese subjects [50]. Women that were diagnosed with estrogen receptor/progesterone receptor positive (ER+/PR+) breast cancers that also expressed high levels of leptin had poorer prognosis than women with low leptin expression [50]. The results from the study of Strong et al. [50] demonstrate that abdominal obesity induces significant changes in the biological properties of ASCs and that these alterations enhance ER+/PR+ breast cancer tumorigenesis through estrogen dependent pathways.

MRI angiography is routinely clinically and experimentally used in the identification of tumor-feeding and -draining vessels, tumor characterization, and treatment planning can be also used with specific contrast agents, for showing morphological structure of angiogenesis in relation vessel permeability [25,40]. Non-invasive quantification of angiogenesis may also be possible with MRI. Moreover, it may include the called four-dimensional (4D) MR angiography, in which high-resolution 3D MRI angiography is combined with dynamic contrast-enhanced MRI [51]. Nowadays, MRI of the breast, thanks to very high sensitivity and specificity, is considered to be a problem solving technique that is able to depict the large majority of physiological and pathological process, in which morphology and vascularity changes are involved and well depicted [52].

## 5. Conclusions

We demonstrated that the use of EF-e-A results in increased graft survival and function in patients that were affected by the outcomes of breast reconstruction and oncoplastic surgery. The authors concluded that the concept of EF-e-A is a reliable alternative to breast implant. In addition, the results obtained suggested that EF-e-A is effective and safe, and that SVFs and ASCs also favour adipose tissue survival. More additional studies are necessary in further evaluating the efficacy of this method.

## Figures and Tables

**Table 1:** Multivariate analysis of the risk of any recurrence (either local or systemic) related to several variables, including the three different surgical approaches.

	Events	Systemic Recurrences	Local Recurrences	OR (95% CI), *p*-Value)
Groups				
Study Group (SG)	4/121 (3.3%)	3/121 (2.5%)	1/121 (0.8%)	0.0456 (0.0075 to 0.2753) *p* = 0.0008
Control Group 1 (CG1)	5/50 (10%)	2/50 (4%)	3/50 (6%)	0.1481 (0.0255 to 0.8604) *p* = 0.0334
Control Group 2 (CG2)	3/7 (43%)	2/7 (29%)	1/7 (15%)	Ref.
Age				
≤50	4/116 (3.5%)	2/116 (1.8%)	2/116 (1.8%)	Ref.
≥50	8/62 (13%)	3/62 (4.8%)	5/62 (8.1%)	4.1481 (1.1963 to 14.3832) *p* = 0.0249
Stage				
0–I	4/93 (4.3%)	1/93 (1.1%)	3/93 (3.2%)	Ref.
II–III	8/85 (9.4%)	4/85 (4.7%)	4/85 (4.7%)	2.3117 (0.6700 to 7.9756) *p* = 0.1848
Lympho-vascular invasion				
No	5/112 (4.5%)	2/112 (1.8%)	2/112 (1.8%)	Ref.
Yes	7/66 (10.7%)	3/66 (4.5%)	5/66 (7.6%)	2.5390 (0.7718 to 8.3530) *p* = 0.1251
Adjuvant or neoadjuvant chemotherapy				
No	4/79 (5.1%)	3/79 (3.7%)	5/79 (6.3%)	Ref.
Yes	8/99 (8.1%)	2/99 (2.0%)	2/99 (2.0%)	1.6484 (0.4777 to 5.6879) *p* = 0.4290
Adjuvant hormone therapy				
No	4/61 (6.6%)	2/61 (3.3%)	2/61 (3.3%)	Ref.
Yes	8/117 (6.9%)	3/117 (2.5%)	5/117 (4.3%)	1.0459 (0.3020 to 3.6223) *p* = 0.9436
Adjuvant radiation therapy				
No	8/149 (5.4%)	3/149 (3.0%)	5/149 (3.3%)	Ref.
Yes	4/29 (14.0%)	2/29 (7.0%)	2/29 (7.0%)	2.8200 (0.7893 to 10.0757) *p* = 0.1105
Body Mass Index				
≤30	3/116 (2.6%)	1/116 (0.8%)	2/116 (1.8%)	Ref.
≥30	9/62 (14.5%)	4/62 (6.4%)	5/62 (8.2%)	6.3962 (1.6635 to 24.5940) *p* = 0.0069

OR = Odds Ratio; CI = Confidence Interval; Ref. = Reference category.

**Table 2:** Mesenchymal derived cell populations characterization in the adipose tissue graft groups.

Cell Population Characterization	Lipoaspirate (A) (cells/mL) *	SVFs Isolation (B) (cells/mL) *	SVFs Enhanced Graft (Lipoaspirate + SVFs) (C) (cells/mL) *	Lipoaspirate Centrifuged (D) (cells/mL) *
Total cells	65,800	13,625,000	427,650	207,750
CD 34+	2829	7,766,250	277,970	149,580
CD 45+	1974	258,8750	85,530	60,247
CD 105+	0	40,8750	21,382	14,542
CD 34+ CD 45+	329	1,635,000	59,870	39,472
CD 105+ CD 45+	0	136,250	12,830	8310
CD 105+ CD34+	0	408,750	17,106	14,542
CD 90+	2632	7,221,250	273,696	124,650
CD 31+	1316	3,133,750	85,530	51,937
CD 31+ CD 45+	5922	953,750	38,488	33,240
Total CD 34+, CD 45-, CD 105+	2500	5,722,500	200,995	110,107

* Values were reported as an average number of every analyzed samples in each group (A). Pure fat harvest lipoaspirate from all patients before any fat managing (CG2); (B). Stromal Vascular Fraction cells (SVFs) suspension sample (alone) from fat graft of five study group (SG) patients underwent Celution® system treatment; (C). SVFs-enhanced fat graft sample from five SG patients before injection; (D). Sample of standard centrifuged fat graft, according to Coleman’s technique, from five CG1 patients.
